# Molecular cloning and subcellular localization of six *HDACs* and their roles in response to salt and drought stress in kenaf (*Hibiscus cannabinus* L.)

**DOI:** 10.1186/s40659-019-0227-6

**Published:** 2019-04-06

**Authors:** Fan Wei, Danfeng Tang, Zengqiang Li, Muhammad Haneef Kashif, Aziz Khan, Hai Lu, Ruixing Jia, Peng Chen

**Affiliations:** 1 Key Laboratory of Plant Genetics and Breeding, College of Agriculture, Guangxi University, Nanning, 530005 People’s Republic of China; 2 Guangxi Key Laboratory of Medicinal Resources Protection and Genetic Improvement, Guangxi Botanical Garden of Medicinal Plants, Nanning, 530025 People’s Republic of China

**Keywords:** Kenaf (*Hibiscus cannabinus* L.), Histone deacetylases, Histone acetylation, Gene expression, Subcellular localization, Stress

## Abstract

**Background:**

Histone acetylation is an important epigenetic modification that regulates gene activity in response to stress. Histone acetylation levels are reversibly regulated by histone acetyltransferases (*HATs*) and histone deacetylases (*HDACs*). The imperative roles of *HDACs* in gene transcription, transcriptional regulation, growth and responses to stressful environment have been widely investigated in *Arabidopsis*. However, data regarding *HDACs* in kenaf crop has not been disclosed yet.

**Results:**

In this study, six *HDACs* genes (*HcHDA2*, *HcHDA6*, *HcHDA8*, *HcHDA9*, *HcHDA19*, and *HcSRT2*) were isolated and characterized. Phylogenetic tree revealed that these HcHDACs shared high degree of sequence homology with those of *Gossypium arboreum*. Subcellular localization analysis showed that GFP-tagged HcHDA2 and HcHDA8 were predominantly localized in the nucleus, HcHDA6 and HcHDA19 in nucleus and cytosol. The HcHDA9 was found in both nucleus and plasma membranes. Real-time quantitative PCR showed that the six *HcHDACs* genes were expressed with distinct expression patterns across plant tissues. Furthermore, we determined differential accumulation of HcHDACs transcripts under salt and drought treatments, indicating that these enzymes may participate in the biological process under stress in kenaf. Finally, we showed that the levels of histone H3 and H4 acetylation were modulated by salt and drought stress in kenaf.

**Conclusions:**

We have isolated and characterized six *HDACs* genes from kenaf. These data showed that *HDACs* are imperative players for growth and development as well abiotic stress responses in kenaf.

**Electronic supplementary material:**

The online version of this article (10.1186/s40659-019-0227-6) contains supplementary material, which is available to authorized users.

## Background

Kenaf (*Hibiscus cannabinus* L.) is a herbaceous non-woody fiber plant which grows mainly in Asia and Africa. The bast fiber composed of 75% cellulose and 7% lignin and offer the advantage of being biodegradable. It has versatile applications such as in paper making, animal bedding, construction, carpet backing, cordage, livestock foraging and biomass crop [1, 2]. Furthermore, kenaf shows high tolerance to saline, alkaline, or arid condition, so it can be used for phytoremediation of saline-alkali soil, or as a drought tolerant crop. However, the mechanism of its tolerance is still unclear.

The basic unit of chromatin is a nucleosome which contains a histone octamer formed with two copies of histone H2A, H2B, H3 and H4. Each histone comprises of a globular domain structure and an unstructured amino-terminal tail extending from the core nucleosome [3, 4]. The N-terminal tail of histone protein can provide sites for diverse post-translational modifications (PTMs), such as acetylation, glycosylation, methylation, ubiquitination, phosphorylation and ADP-ribosylation. Histone acetylation is one of the well-characterized PTMs [5, 6] and an important epigenetic modification that regulates gene activity in response to stress. Acetylation state of the ε-amino group of conserved lysine residues within all four core histones was reversibly regulated by the activities of histone acetyltransferases (*HATs*) and histone deacetylases (*HDACs*) [7, 8]. In short, *HDACs* acted in concert with *HATs* to regulate dynamic and reversible histone acetylation, which modified chromatin structure and function thus affected gene transcription resulting in the regulation of multiple cellular processes including stress response.

*HDACs* were sorted into different families and were generally conserved in fungi and eukaryotes, including yeast, animals and higher plants. Plant *HDACs* were categorized into three families, RDP3/HDA1 and SIR2 families, which were homologous to *HDACs* found in yeast and animals, and *HD2* family, which was unique to plants [9]. Many plants *HDACs* have been cloned and identified as transcriptional activators or repressors in various biological processes [9–20]. In *Arabidopsis*, *AtHDACs* play important roles in seed development, germination, seed dormancy, circadian regulation, hypocotyls growth, female gametophyte development, embryogenesis, root hair development, leaf morphogenesis, flower development, responses to day length, environmental stresses and defensive response against pathogen attack [21–29]. Although *HDACs* are thought to play imperative roles in plant growth, developmental processes, and responses to stressful conditions, little is known about the biological functions of *HDAC* genes in kenaf.

In this study, we identified and analyzed the characteristics of six *HDAC* coding genes, including *HcHDA2*, *HcHDA6*, *HcHDA8*, *HcHDA9*, *HcHDA19* and *HcSRT2* from kenaf. Subcellular location of HcHDACs was confirmed by GFP-tagged transient expression assays with tobacco protoplasts. In addition, tissue-specific and stress-responsive expression patterns of the six *HcHDAC* genes were evaluated by qRT-PCR analysis. Furthermore, the histone H3 and H4 acetylation levels of kenaf roots were analyzed under salt and drought treatments. These data will provide the foundation for further research on the function of *HcHDACs* in growth, development, and responses to abiotic stress of kenaf.

## Materials and methods

### Plant materials and growth conditions

The plant materials were grown at Guangxi University experimental farm (located at 108°22′E, 22°48′N). Seeds of kenaf cultivar P3B were raised on loamy soil with pH = 6.9 under natural conditions. Roots, stems and leaves were sampled at 7-day-old seedling and anthesis stages, and anthers were collected at tetrad, mononuclear, and dual-core stages for cloning and expression pattern analysis. Fresh samples were immediately frozen in liquid nitrogen and stored at − 80 °C for further analysis. For the stress treatments, 7-day-old seedlings were transplanted to hydroponic culture and exposed to different salt (0, 100, and 200 mM NaCl) and drought (0%, 10, and 20% PEG6000) stress levels. The culture condition was configured at light/dark cycle of 14/10 h at 28 °C with 60% relative humidity. After exposure to these stresses for 7 days, roots were sampled and immediately frozen in liquid nitrogen for further gene expression and protein immunoblotting experiments.

### PCR amplification and cloning of *HcHDACs*

Total genomic DNAs were extracted from the plant samples using FastPure™ Plant DNA Isolation Mini Kit (Vazyme, DC104) while RNAs were isolated by FastPure™ Plant RNA Isolation Mini Kit (Vazyme, RC401) according to the manufacturer’s protocol. cDNAs were synthesized from total RNA using the HiScript^®^ II One Step RT-PCR Kit (Vazyme, P611). The DNA and cDNA sequences of six *HcHDAC* genes were cloned by homology cloning according to the RNA-seq data of kenaf [30]. Gene-specific primers were designed by Primer Premier 5 software and are shown in Additional file 1: Table S1. The PCR amplification was performed in the following configuration: an initial denaturation at 95 °C for 3 min, followed by 35 cycles of denaturation at 95 °C for 15 s, annealing at 56–65 °C for 15 s, 72 °C extension for 1–3 min and final extension at 72 °C for 5 min. All PCR reactions were carried out using 25 μL reaction system; 12.5 μL 2 × Phanta Max Master Mix (Vazyme, China), 1 μL forward primer, 1 μL reverse primer, and 1 μL DNA/cDNA template. PCR products were recycled and then sequenced in BGI (China).

### DNA and protein sequence analysis

Physicochemical property of HcHDACs was predicted by ProtParam tool online (http://web.expasy.org/protparam/). Gene exon–intron structures were analyzed using the Gene Structure Display Server (GSDS2.0, http://gsds.cbi.pku.edu.cn/) [31] by comparing the codon sequences and genomic sequences of *HcHDACs.* The conserved domains were predicted with Pfam program (http://pfam.janelia.org/) and HMMER-based SMAT Website (http://smart.embl-heidelberg.de/). The domain architecture was drawn using DOG2.0 software (http://gsds.cbi.pku.edu.cn/). HcHDAC proteins along with proteins from *Arabidopsis thaliana*, *Gossypium arboretum, Oryza sativa Japonica Group*, and *Solanum lycopersicum* downloaded from the National Center for Biotechnology Information (NCBI) databases (https://www.ncbi.nlm.nih.gov/) were aligned with Clustal X [32]. The phylogenetic tree was constructed by the neighbor-joining (NJ) method in MEGA (version 5.0) software [33]. The stability of the internal nodes was assessed by bootstrap analysis of 1000 replicates.

### Protoplast transient expression analysis

A homologous recombination method was used to construct transient expression vectors pBWA(V)HS-HcHDA2-GLosgfp, pBWA(V)HS-HcHDA6-GLosgfp, pBWA(V)HS-HcHDA8-Glosgfp, pBWA(V)HS-HcHDA9-GLosgfp and pBWA(V)HS-HcHDA19-GLosgfp. Tobacco leaf mesophyll protoplasts were isolated from fully expanded leaves of 8-week-old plants [34]. Twenty micrograms of each GFP fusion plasmid was cotransfected into 200 μL protoplasts (4 × 10^4^ protoplasts) using PEG–calcium transfection solution, respectively. Protoplasts were incubated at 25 °C overnight to allow expression of the introduced genes. The GFP fluorescence was examined and photographed using a Leica SP8 confocal fluorescence microscope (Leica, Wetzlar, Germany).

### qRT-PCR analysis

qRT-PCR was performed with ChamQ™ SYBR^®^ qPCR Master mix (Vazyme, Q311) using Bio-Rad CFX96 Real-Time PCR Detection System. The gene-specific primers for qRT-PCR were designed by Primer Premier 5 software and are shown in Additional file 1: Table S1. The reaction conditions were as follows: 95 °C for 1 min, followed by 50 cycles of 95 °C for 10 s, and 60 °C for 30 s. The kenaf *H3*, *ACT3* and *18S* genes were used as internal controls for normalizing gene expression levels. Each gene contains triplicate for qRT-PCR. The comparative CT value method [35] was employed to analyze the expression profiles of *HcHDACs*.

### Western blotting

Kenaf roots sampled from control, NaCl and drought treated seedlings were ground to power and the histones were extracted according to the manufacturer’s protocol (BB31171, BestBio, Shanghai, China), Around 50 μg histone of each sample was separated by 15% SDS-PAGE gels and transferred to a polyvinylidine fluoride fluoropolymer (PVDF) membrane (0.45 μm, Millipore, Darmstadt, Germany) using Trans-Blot system (Bio-Rad, California, USA). 5% skim milk powder was used to block the membranes in TBST buffer (20 mM Tris–HCl, 150 mM NaCl, 0.05% Tween 20) for 1 h at room temperature. The target protein bands were sequentially detected by Anti-Histone H3 (acetyl K9) Antibody (1:1000 dilution in TBST) (ab12179, abcam, UK), Anti-Histone H3 (acetyl K27) Antibody (1:1000 dilution in TBST) (ab4729, abcam, UK), Anti-Histone H4 (acetyl K5) Antibody (1:50,000 dilution in TBST) (ab51997, abcam, UK) and Anti-Histone H3 Antibody (1:1000 dilution in TBST) (ab1791, abcam, UK). Alkaline Phosphatase Goat anti-Rabbit IgG (H + L) (ZB-2308, CWBIO, Beijing, China) and Alkaline Phosphatase Horse anti-Mouse IgG (H + L) (ZB-2310, CWBIO, Beijing, China) were used as secondary antibodies. Last, an enhanced chemiluminescence (ECL) immunoblotting detection kit (P90719, Millipore, USA) was used for signal detection. The experiments were carried out three times and the Image J software was used to quantify the relative protein levels.

## Results

### Cloning and identification of kenaf histone deacetylases

**Fig. 1:**
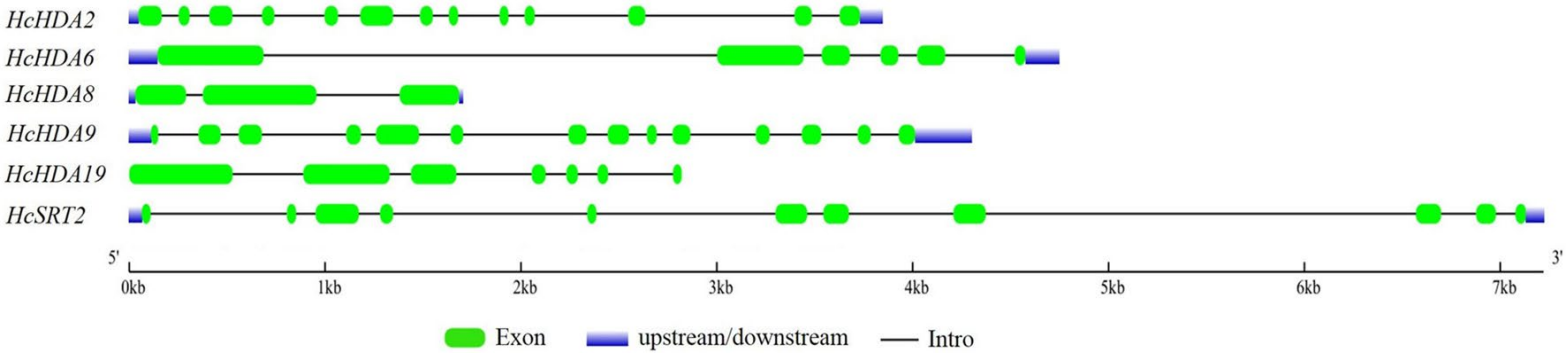
The exon–intron structure of kenaf HcHDACs. The lengths and positions of introns and exons are shown on the figure. The green boxes and gray lines denote exons and introns, respectively

Six kenaf histone deacetylases genes, including *HcHDA2*, *HcHDA6*, *HcHDA8*, *HcHDA9*, *HcHDA19* and *HcSRT2* were cloned and identified. These *HcHDAC* genes contained a complete open reading frame (ORF) ranged from 1080 to 1428 bp and their protein length varied from 359 to 475 amino acids (aa). The molecular weights and isoelectric points of these HcHDACs ranged from 39.9 to 62.08 kDa and 5.05 to 9.43, respectively. The GRAVY (grand average of hydropathicity) results indicated that all these HcHDACs were hydrophilic (Table 1). In addition, the DNA and cDNA sequences of these *HcHDAC* genes were analyzed to confirm the intro-exon organization. The data showed that their conserved coding regions contained various numbers of exons (Table 1 and Fig. 1).

**Table 1:** Histone deacetylases identified in kenaf

*HDAC* families	Gene name	Accession number^a^	ORF length (bp)^b^	Protein length^c^	MW (kDa)	pI	GRAVY	Number of exons	Localization^d^
RPD3/HDA1	HcHDA2	MH732953	1080	359	39.90	8.51	− 0.157	13	Nucleus
	HcHDA6	MH732954	1413	470	52.68	5.29	− 0.507	6	Nucleus
	HcHDA8	MH732955	1143	380	41.04	5.30	− 0.121	3	Nucleus
	HcHDA9	MH732958	1290	429	49.09	5.05	− 0.399	14	Nucleus, cytosol
	HcHDA19	MH732956	1428	475	53.35	5.45	− 0.436	7	Nucleus, cytosol
Sirtuin	HcSRT2	MH732957	1158	385	42.83	9.43	− 0.262	11	Chloroplast

MW, molecular weight; pI, isoelectric point; GRAVY, grand average of hydropathicity

^a^Accession numbers of full-length protein sequence available at NCBI (http://www.ncbi.nlm.nih.gov/)

^b^Length of open reading frame (number of base pair)

^c^Length of protein (number of amino acid)

^d^Subcellular Localization of kenaf histone deacetylases supported by Plant-mPLoc (http://www.csbio.sjtu.edu.cn/bioinf/plant-multi/)

### Phylogenetic and domain architecture analysis of kenaf histone deacetylases

**Fig. 2:**
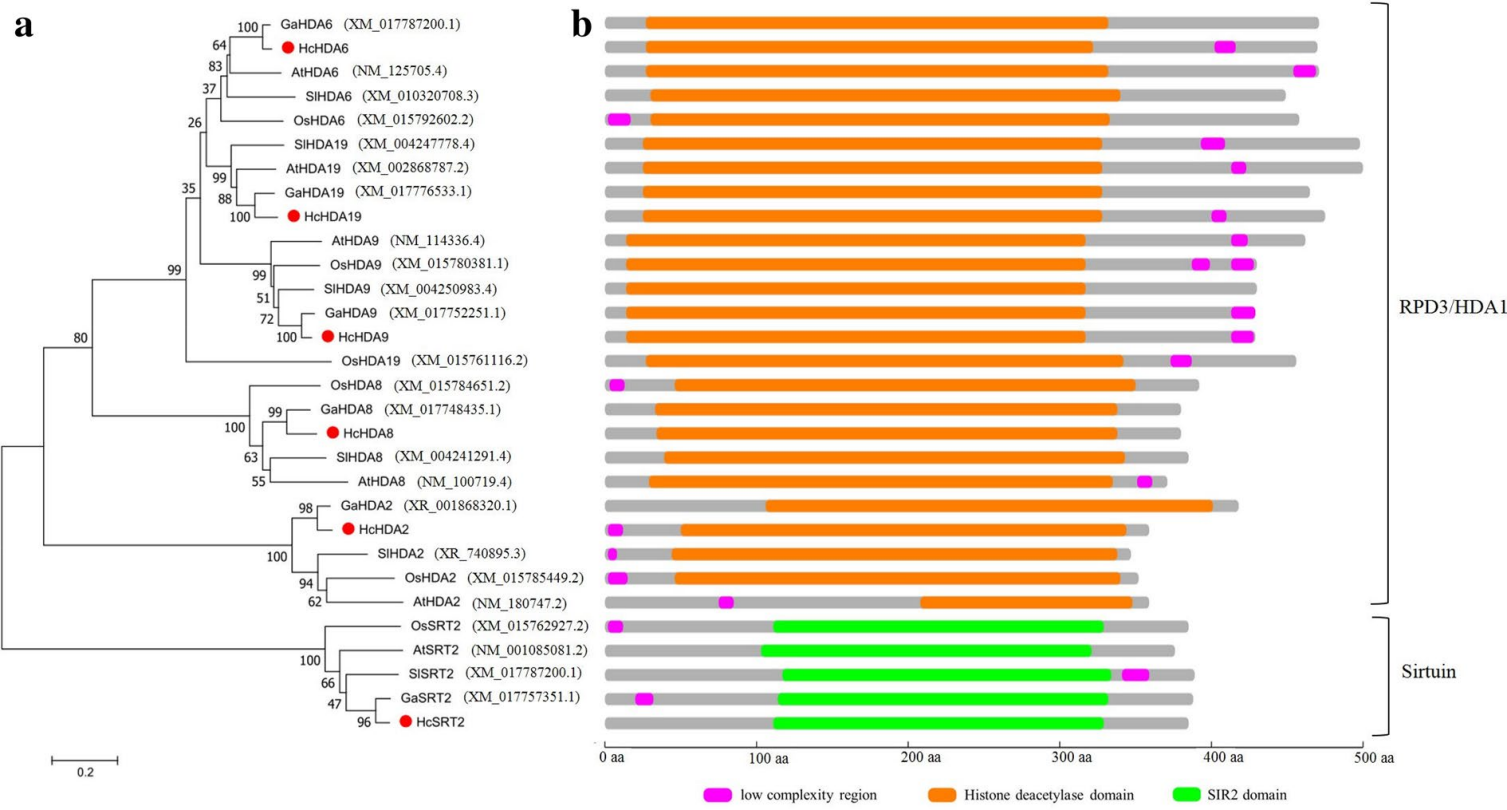
Phylogenetic analysis and domain organization of HcHDACs in kenaf. **a** Phylogenetic tree of HDACs from kenaf and other plants. **b** Domain architecture of HDACs from kenaf and other plants. The phylogenetic tree was generated using MEGA 5.0 software and the numbers at the nodes indicate the bootstrap values (bootstrap values > 50% were shown). The GenBank accession numbers of the sequences used were noted in brackets. *At*, *Arabidopsis thaliana; Ga*, *Gossypium arboreum*; *Os, Oryza sativa Japonica Group; Sl*, *Solanum lycopersicum.* Different domains are represented by different colors and lengths at their precise position in the protein sequence from the N-terminus to the C-terminus. The proteins belonging to each family were grouped together

To reveal the evolutionary relationship of histone deacetylases in kenaf, a phylogenetic tree was constructed using histone deacetylases from *Arabidopsis thaliana*, *Gossypium arboreum*, *Oryza sativa Japonica Group* and *Solanum lycopersicum*. Phylogenetic analysis was performed using MEGA 5.0 software based on the deduced amino acid sequences of these histone deacetylases. The neighbor-joining phylogenetic tree showed that HDACs from kenaf and *Gossypium arboreum* were at the same clade and showed a strong relationship with high degree of similarity, especially, HcHDA6 and GaHDA6, HcHDA9 and GaHDA9, HcHDA19 and GaHDA19 were grouped together with strong boot-strap support (100%), respectively (Fig. 2a). By analysis of domain architecture, HcHDA2, HcHDA6, HcHDA8, HcHDA9 and HcHDA19 contained a typical deacetylase catalytic domain and belonged to RPD3/HDA1 subfamily, while HcSRT2 contained the conserved domain SIR2 and belonged to Sirtuin subfamily (Fig. 2b).

### Subcellular localization of HcHDACs

**Fig. 3:**
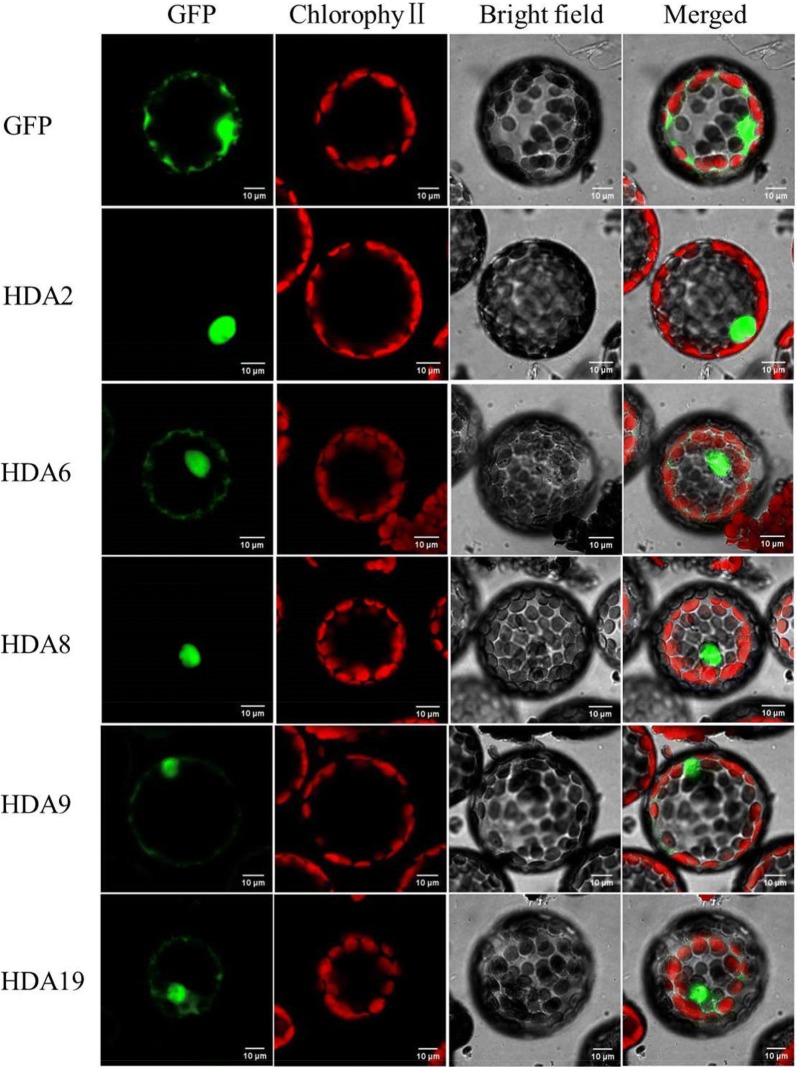
Protoplast transient expression analysis using vectors with GFP fusion. Subcellular location of *HcHDA2*, *HcHDA6*, *HcHDA8*, *HcHDA9* and *HcHDA19* were determined via tobacco protoplast PEG transfection using vectors with GFP fusion, respectively. Red color indicated autofluorescence emitted by chloroplasts

Plant-mPLoc was used to determine the possible localization sites of HcHDACs. Interestingly, HcHDACs were predicted to have different subcellular localizations including nucleus, cytosol and chloroplasts (Table 1), which implied that they might have distinct roles in kenaf. To further determine the subcellular location of HcHDACs, full-length cDNAs were fused to *Green Fluorescent Protein* (*GFP*) driven by CaMV 35S promoter and transiently expressed in protoplasts of tobacco suspension culture cells. As shown in Fig. 3, HcHDA2 and HcHDA8 were localized in the nucleus and HcHDA19 was localized in the nucleus and cytosol, which were consistent with the predicted location using bioinformatics program. Whereas, HcHDA9 was localized in both the nucleus and plasma membrane, which was different from the predicted location data. HcHDA6 was not only localized in the nucleus as predicted by Plant-mPLoc programs, but also in the cytosol. The cytosolic localization of HcHDA6 and HcHDA19 suggested that they might play a vital catalytic role on diverse proteins outside the nucleus other than in histone acetylation.

### The expression pattern of *HDAC* genes in different tissues at various developmental stages of kenaf

**Fig. 4:**
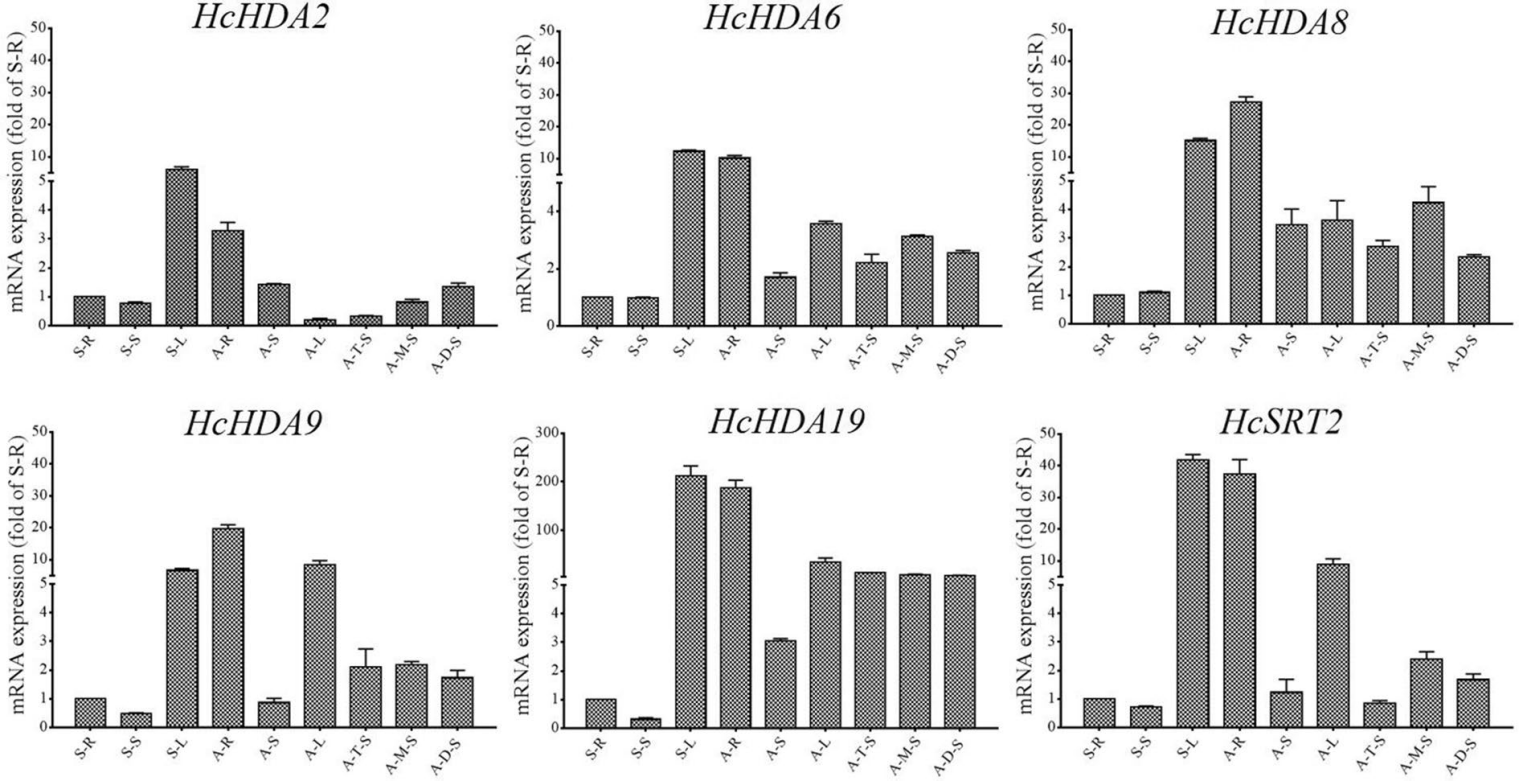
The expression analysis of *HcHDAC* genes in kenaf different tissues at various developmental stages by qRT-PCR. S-R, seedling root; S-S, seedling stem; S-L, seedling leaf; A-R, anthesis root; A-S, anthesis stem; A-L, anthesis leaf; A-T, anther tetrad stage; A-M, anther mononuclear stage; A-D, anther dual-core stage. Quantitative RT-PCR was performed using gene specific primers. Data are the mean ± SEM of three independent experiments

Histone deacetylases are essential for plant growth and development. To determine the detail expression patterns of these six *HDAC* genes in different tissues at various developmental stages of kenaf, real-time qPCR analysis was conducted. Tissue-specific gene expression was normalized to the gene expression levels in the root from seeding stage. All genes detected in the four tissues (root, stem, leaf and anther) are presented in Fig. 4. All the six *HcHDAC* genes showed high expression levels in leaf at the seedling stage and root at anthesis stage. Except for *HcHDA9*, which was up-regulated both in root, stem and leaf, the othe five *HcHDAC* genes were up-regulated in root and stem, but down-regulated in leaf with kenaf development. Among the six *HcHDAC* genes, *HcHDA19* exhibited the greatest expression differences in leaf of seedling and root of anthesis, 200-fold increase was detected compared with control.

Flowering is vital for plants to complete the life cycle and reproduce offspring. To investigate the expression of these six *HcHDAC* genes during kenaf anther development, samples from three anther growth stages were used including tetrad (A–T), mononuclear (A–M) and dual-core stage (A–D). Except for *HcHDA19*, which showed no significant differences during anther development, the other five *HcHDAC* genes expression changed with anther development. The expression of *HcHDA2* increased gradually with anther growth. *HcHDA6*, *HcHDA8*, *HcHDA9* and *HcSRT2* exhibited consistent expression patterns, which were up-regulated at mononuclear stage and then down-regulated at dual-core stage (Fig. 4).

### *HcHDACs* are involved in salt and drought stress responses

**Fig. 5:**
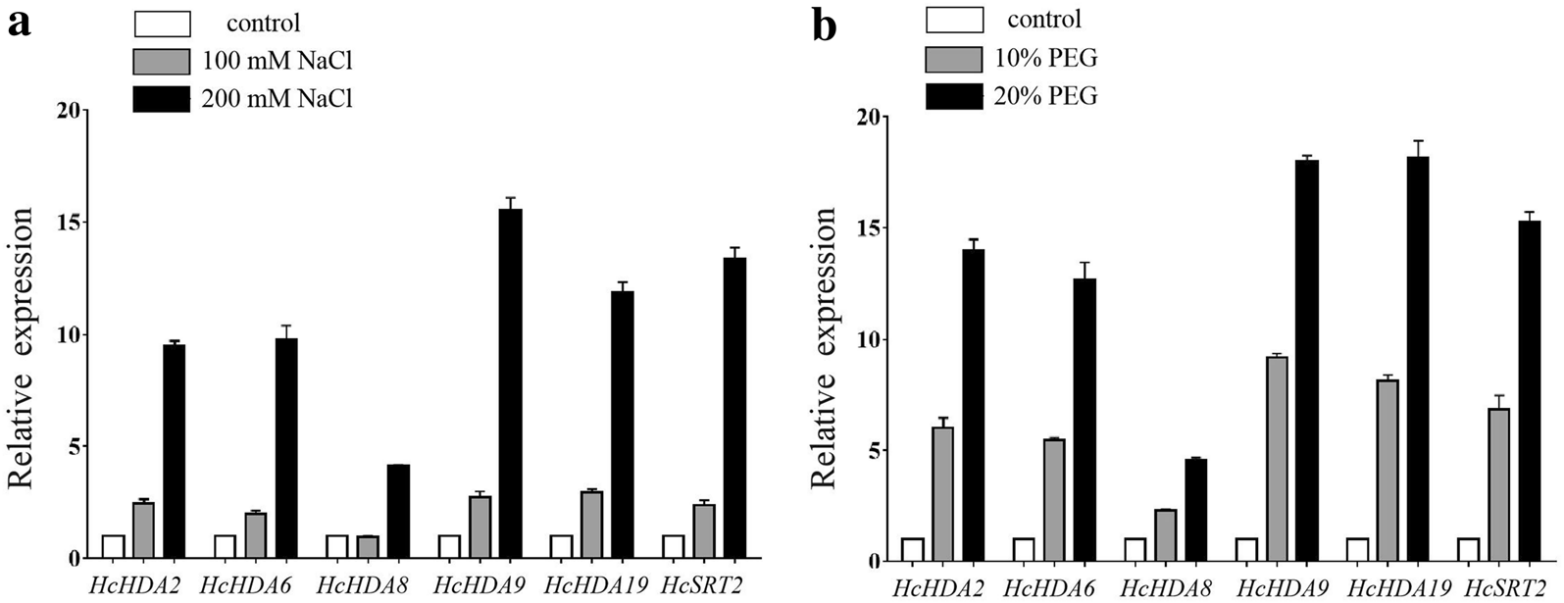
Expression profiles of *HcHDAC* genes under NaCl and PEG conditions. 7-day-old plants were treated with 0, 100, 200 mM NaCl, and 0%, 10%, 20% PEG6000 for 7 days and the roots were harvested for qRT-PCR analysis of gene expression. The x-axis presents different genes. The y-axis shows expression levels relative to the control, which was set to 1.0. Data are the mean ± SEM of three independent experiments

**Fig. 6:**
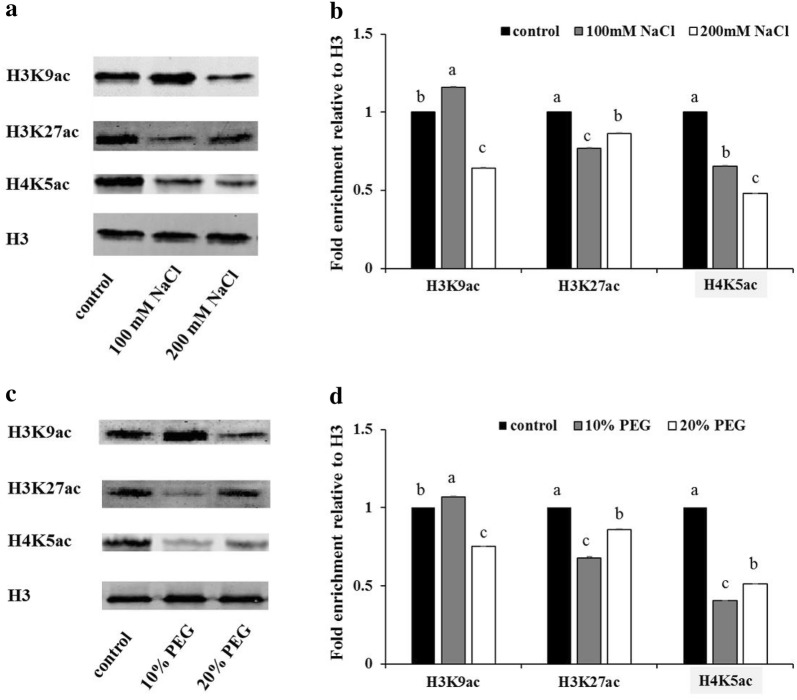
Levels of histone H3K9ac, H3K27ac and H4K5ac under NaCl and drought treatments. **a**, **c** Western blot showing the H3K9ac, H3K27ac and H4K5ac status in kenaf roots treated with NaCl and PEG solution. 7-day-old seedlings were treated under 0, 100, 200 mM NaCl, and 0%, 10%, 20% PEG6000 conditions for 7 days, respectively, and the roots were sampled for histone proteins extraction. **b**, **d** Quantification of western blot results. Signal intensities were measured using the ImageJ software and normalized to the loaded amount of H3. Values are expressed as fold change over control treatment. Shown is the mean ± SEM of three independent experiments. *P* value < 0.05, Student’s t-test

Evidence suggests that *HDACs* participate in various abiotic stress responses in plants. For further study the potential functions of these six *HcHDACs* under abiotic stress in kenaf, qRT-PCR was used to determine the relative mRNA abundance under salt and drought stress. Salt stress significantly induced the six *HcHDAC* genes expression (Fig. 5a). Except for *HcHDA8*, the expression of the other five *HcHDAC* genes were significantly increased with the increasing concentrations of NaCl, which showed two- to threefold increases under 100 mM and 10- to 15-fold increases under 200 mM NaCl treatment, comparison with control. Substantially changes in the expression of these *HcHDAC* transcripts were observed in *HcHDA9* under 200 mM NaCl treatment, this resulted in 15-fold increase, compared with control. An exposer to drought, the expression patterns of the six *HcHDAC* genes showed similar trend to salt stress. Except for *HcHDA8*, the other five *HcHDAC* genes were strongly induced after PEG treatment (Fig. 5b). In addition, status of H3 and H4 acetylation following NaCl and PEG treatments was analyzed. As shown in Fig. 6, the level of H3K9ac increased under 100 mM NaCl treatment but decreased under 200 mM NaCl treatment. Status of H3K27ac and H4K5ac all decreased after NaCl treatments (Fig. 6a, b). High levels of H3K9ac and low levels of H3K27ac and H4K5ac were observed following 10% PEG treatment. Meanwhile, the level of H3K9ac, H3K27ac and H4K5ac were all down-regulated in response to 20% PEG stress (Fig. 6c, d).

## Discussion

### Characterization and expression patterns of kenaf histone deacetylases

A greater number of *HDAC* genes have been cloned, identified and characterized in various plant species as well the functions of certain *HDACs* have been investigated [25, 27]. *Arabidopsis* genome encodes eighteen *HDACs* [9, 10]. The rice (*Oryza sativa* L.) genome contains eighteen *HDACs* [14, 15]. Fifteen *HDACs* were characterized in tomato (*Solanum lycopersicum*) [18]. Eleven *HDACs* were analyzed from litchi (*Litchi chinensis Sonn*) [20]. *HDACs* were also identified in maize (*Zea mays*), poplar (*Populus trichocarpa*) and banana, each contained fifteen, sixteen and seventeen members, respectively [16, 36, 37]. Compared to the aforementioned plants, hindered by a lack of genomic information, relatively few *HDACs* were characterized in kenaf. Only six *HcHDAC* genes were cloned and identified using the molecular biology and bioinformatics analysis in the present study (Table 1), the other *HcHDAC* genes in kenaf need to be identified and characterized in the future study.

In *Arabidopsis*, HDA8 was reported to localize in the cytosol [38], while HDA6 and HDA19 in the nucleus [23, 39, 40]. In the present study, we found that HcHDA8 was localized in the nucleus, which was different from AtHDA8. HcHDA6 and HcHDA19 were not only localized in the nucleus but also in the cytosol, suggesting a possible shuttling process between the cytoplasm and the nucleus (Fig. 3). It should be noted, however, that the subcellular localization assays in the present study were performed using tobacco cells, and therefore, the localization pattern in situ could be influenced by kenaf-specific interactions.

The expression of the six *HcHDAC* genes was detectable in roots, stems, leaves and anthers (Fig. 4). The different expression pattern of these *HcHDAC* genes may imply different functions in growth and development of kenaf. However, the further research on specific roles of these genes in kenaf was necessary. Flowering is vital for plants to complete the life cycle and reproduce offspring. Flower growth and development is a tightly and exclusively regulated process and a number of histone acetylation factors have been identified to be associated with flower organ formation and flowering time control [27, 41]. In *Arabidopsis*, there were a variety of flower developmental aberrations observed in the loss-of-function *AtHDA19* line, including reduced female fertility, smaller siliques and abnormal flowers [39, 42, 43]. The dysfunction of *AtGCN5* caused short stamens and petals, and defects in floral organ identity [44]. *MCC1* over-expression mutant led to meiotic defects resulting in abortion in about half of the male and female gametes due to histone hyperacetylation [12]. *HAM1* and *HAM2* played important roles in gametogenesis redundantly by genetic and cytological analysis [45]. In this work, the expression of five *HcHDAC* genes, including *HcHDA2*, *HcHDA6*, *HcHDA8*, *HcHDA9* and *HcSRT2* changed with anther development. A possible function of these *HcHDACs* involved in kenaf anther growth was suggested.

### Effects of abiotic stresses on *HcHDACs* expression in kenaf

Kenaf can be used in the phytoremediation of salt contaminated soil and as a drought tolerant crop. Though researches on kenaf salt and drought tolerance have made some progresses [46–52], the mechanisms of salt and drought tolerance are still unclear. Histone acetylation is an important epigenetic modification, which regulates gene activity in response to stress. Till now, there is no reports addressing histone acetylation modification in kenaf. Therefore, it is necessary to explore the mechanism of salinity and drought tolerance from the aspect of histone acetylation modification. Researches showed histone acetylation was involved in plant responses to salt and drought stresses [53]. In *Arabidopsis*, the expression levels of histone deacetylases *HD2A*, *HD2B*, *HD2C* and *HD2D* were suppressed by high salt treatment [54]. The overexpression of *HD2C* and *HD2D* showed enhanced tolerance to drought and salt [55, 56]. *HDA6* was associated with *HDT3*/*HD2C* regulated gene expression in response to salt stress and also involved in drought stress tolerance by regulating gene expression in acetate biosynthesis pathway [57, 58]. *SlHDACs* were induced in various degrees under high salinity and dehydration in tomato (*Solanum lycopersicum*) [53].

To understand whether and how kenaf responses to environmental stress by epigenetics, the expression of *HcHDACs* following treatments with NaCl and PEG solution was analyzed in our study. qRT-PCR results showed that these six *HcHDAC* genes were all dramatically induced in various degrees under salt and drought treatments (Fig. 5a, b), indicating that these *HcHDAC* genes were involved in the epigenetic regulation of salt and drought resistance genes. Furthermore, we showed that NaCl and PEG treatments can influence the levels of histone H3 and H4 acetylation, indicating that histone acetylation may play a key role in the response to both NaCl and PEG stress. One or more of the *HcHDAC* genes may hold promise for improving stress tolerance in kenaf via genetic engineering. The knowledge about *HcHDACs* in salt and drought responses will contribute to further understanding of molecular mechanisms that control salt and drought stress responses, and how *HDACs* function in this process. We hoped that this would eventually lead to a better understanding of how plants adapt to environmental changes and a long-term improvement of salt and drought stress tolerance.

## Conclusion

Histone acetylation and deacetylation, which are regulated by histone acetyltransferases (*HATs*) and histone deacetylases (*HDACs*), are key players in the modification of chromatin structure and regulation of gene expression. However, data regarding *HDACs* in kenaf crop has not been disclosed yet. In the present study, we isolated and characterized six *HDACs* genes from kenaf. Phylogenetic tree revealed that HcHDACs shared high degree of sequence homology with those of *Gossypium arboreum*. Transient expression of tobacco protoplasts showed that HcHDA2 and HcHDA8 were localized in the nucleus, HcHDA6 and HcHDA19 in nucleus and cytosol, while HcHDA9 in nucleus and plasma membranes. Six *HcHDACs* genes were expressed with distinct expression patterns in different tissues examined and all of them were salt and drought stress-responsive. Furthermore, our data showed that histone acetylation levels were affected under salt and drought stress treatment. It is suggested that *HDACs* are imperative players for growth and development as well stress responses in kenaf. Further studies are required to confirm the functions of these genes as well as to explore the mechanisms that underlie responses to salt and drought stress in kenaf.

## Additional file

**Additional file 1: Table S1.** Description of primers used in the study.

## Abbreviations

*HDACs*: histone deacetylases
*HATs*: histone acetyltransferases
PTMs: post-translational modifications
qRT-PCR: quantitative real time-PCR
MW: molecular weight
pI: isoelectric point
GRAVY: grand average of hydropathicity
ORFs: open reading frames
GFP: Green Fluorescent Protein

## References

[1] Danalatos NG, Archontoulis SV (2010). Growth and biomass productivity of kenaf (*Hibiscus cannabinus*, L.) under different agricultural inputs and management practices in central Greece. Ind Crop Prod..

[2] Ramesh M (2016). Kenaf (*Hibiscus cannabinus* L.) fibre based bio-materials: a review on processing and properties. Prog Mater Sci.

[3] Luger K, Mader AW, Richmond RK (1997). Crystal structure of the nucleosome core particle at 28 angstrom resolution. Nature..

[4] Campos EI, Reinberg D (2009). Histones: annotating chromatin. Ann Rev Genet.

[5] Allfrey VG, Faulkner R, Mirsky AE (1964). Acetylation and methylation of histones and their possible role in the regulation of RNA synthesis. Proc Natl Acad Sci USA.

[6] Lusser A, Kolle D, Loidl P (2001). Histone acetylation: lessons from the plant kingdom. Trends Plant Sci.

[7] Brownell JE, Zhou JX, Ranalli T (1996). Tetrahymena histone acetyltransferase A: a homolog to yeast Gcn5p linking histone acetylation to gene activation. Cell.

[8] Nagy L, Kao HY, Chakravarti D (1997). Nuclear receptor repression mediated by a complex containing SMRT, mSin3A, and histone deacetylase. Cell.

[9] Pandey R, Muller A, Napoli CA (2002). Analysis of histone acetyltransferase and histone deacetylase families of *Arabidopsis thaliana* suggests functional diversification of chromatin modification among multicellular eukaryotes. Nucleic Acids Res.

[10] Perrella G, Consiglio MF, Aiese-Cigliano R (2010). Histone hyperacetylation affects meiotic recombination and chromosome segregation in *Arabidopsis*. Plant J..

[11] Lagace M, Chantha SC, Major G (2003). Fertilization induces strong accumulation of a histone deacetylase (HD2) and of other chromatin-remodeling proteins in restricted areas of the ovules. Plant Mol Biol.

[12] Demetriou K, Kapazoglou A, Tondelli A (2009). Epigenetic chromatin modifiers in barley: I. Cloning, mapping and expression analysis of the plant specific HD2 family of histone deacetylases from barley, during seed development and after hormonal treatment. Physiol Plant..

[13] Busconi M, Reggi S, Fogher C (2009). Evidence of a sirtuin gene family in grapevine (*Vitis vinifera* L.). Plant Physiol Biochem..

[14] Hu YF, Qin FJ, Huang LM (2009). Rice histone deacetylase genes display specific expression patterns and developmental functions. Biochem Bioph Res Comm.

[15] Liu X, Luo M, Zhang W (2012). Histone acetyltransferases in rice (*Oryza sativa* L.): phylogenetic analysis, subcellular localization and expression. Bmc Plant Biol..

[16] Hu Y, Zhang L, Zhao L (2011). Trichostatin A selectively suppresses the cold-induced transcription of the ZmDREB1 gene in maize. PLoS ONE.

[17] Bourque S, Dutartre A, Hammoudi V (2011). Type-2 histone deacetylases as new regulators of elicitor-induced cell death in plants. New Phytol.

[18] Aiese-Cigliano R, Sanseverino W, Cremona G (2013). Genome-wide analysis of histone modifiers in tomato: gaining an insight into their developmental roles. Bmc Genomics.

[19] Zhao LM, Lu JX, Zhang JX (2015). Identification and characterization of histone deacetylases in tomato (*Solanum lycopersicum*). Front Plant Sci..

[20] Peng MJ, Ying PY, Liu XC (2017). Genome-wide identification of histone modifiers and their expression patterns during fruit abscission in Litchi. Front Plant Sci..

[21] Xu CR, Liu C, Wang YL (2005). Histone acetylation affects expression of cellular patterning genes in the *Arabidopsis* root epidermis. Proc Natl Acad Sci USA.

[22] Hollender C, Liu ZC (2008). Histone deacetylase genes in *Arabidopsis* development. J Integr Plant Biol.

[23] Wu K, Zhang L, Zhou CY (2008). HDA6 is required for jasmonate response, senescence and flowering in *Arabidopsis*. J Exp Bot.

[24] Tian L, Wang JL, Fong MP (2003). Genetic control of developmental changes induced by disruption of *Arabidopsis* histone deacetylase 1 (AtHD1) expression. Genetics.

[25] Yu CW, Liu XC, Luo M (2011). Histone deacetylase6 Interacts with flowering LOCUS D and regulates flowering in *Arabidopsis*. Plant Physiol.

[26] Ma XJ, Lv SB, Zhang C (2013). Histone deacetylases and their functions in plants. Plant Cell Rep.

[27] Liu XC, Yang SG, Zhao ML (2014). Transcriptional repression by histone deacetylases in plants. Mol Plant..

[28] Wang Z, Cao H, Chen F (2014). The roles of histone acetylation in seed performance and plant development. Plant Physiol Biochem.

[29] Ma X, Zhang C, Zhang B (2016). Identification of genes regulated by histone acetylation during root development in *Populus trichocarpa*. Bmc Genomics..

[30] Chen P, Ran SM, Li R (2014). Transcriptome de novo assembly and differentially expressed genes related to cytoplasmic male sterility in kenaf (*Hibiscus cannabinus* L.). Mol Breed.

[31] Hu B, Jin JP, Guo AY (2015). GSDS 2.0: an upgraded gene feature visualization server. Bioinformatics..

[32] Thompson JD, Higgins DG, Gibson TJ (1994). CLUSTAL W: improving the sensitivity of progressive multiple sequence alignment through sequence weighting, position-specific gap penalties and weight matrix choice. Nucleic Acids Res.

[33] Tamura K, Peterson D, Peterson N (2011). MEGA5: molecular evolutionary genetics analysis using maximum likelihood, evolutionary distance, and maximum parsimony methods. Mol Biol Evol.

[34] Wu H, Liu WM, Tu Q (2011). Culture and chemical-induced fusion of tobacco mesophyll protoplasts in a microfluidic device. Microfluid Nanofluid.

[35] Livak KJ, Schmittgen TD (2001). Analysis of relative gene expression data using real-time quantitative PCR and the 2(T)(-Delta Delta C) method. Methods.

[36] Li S, Zhang C, Zhang Y (2015). Sequence and gene expression of histone deacetylases (HDAC) gene family of *Populus trichocarpa*. J Northwest A F Univ Nat Sci Ed.

[37] Han YC. Histone deacetylases are involved in ERF-mediated transcriptional regulation of banana fruit ripening: South China Agricultural University; 2016.

[38] Alinsug MV, Chen FF, Luo M (2012). Subcellular localization of class II HDAs in *Arabidopsis thaliana*: nucleocytoplasmic shuttling of HDA15 is driven by light. PLoS ONE.

[39] Zhou CH, Zhang L, Duan J (2005). Histone deacetylase19 is involved in jasmonic acid and ethylene signaling of pathogen response in *Arabidopsis*. Plant Cell..

[40] Earley K, Lawrence RJ, Pontes O (2006). Erasure of histone acetylation by *Arabidopsis* HDA6 mediates large-scale gene silencing in nucleolar dominance. Genes Dev.

[41] Rolland-Lagan AG, Bangham JA, Coen E (2003). Growth dynamics underlying petal shape and asymmetry. Nature.

[42] Tian L, Chen ZJ (2001). Blocking histone deacetylation in *Arabidopsis* induces pleiotropic effects on plant gene regulation and development. Proc Natl Acad Sci USA.

[43] Tian L, Fong MP, Wang JJ (2005). Reversible histone acetylation and deacetylation mediate genome-wide, promoter-dependent and locus-specific changes in gene expression during plant development. Genetics.

[44] Vlachonasios KE, Thomashow MF, Triezenberg SJ (2003). Disruption mutations of ADA2b and GCN5 transcriptional adaptor genes dramatically affect *Arabidopsis* growth, development, and gene expression. Plant Cell..

[45] Latrasse D, Benhamed M, Henry Y (2008). The MYST histone acetyltransferases are essential for gametophyte development in *Arabidopsis*. BMC Plant Biol.

[46] Lan T, Wang B, Tong ZJ (2007). Identification research on drought tolerance of Kenaf germplasm resources. Plant fiber sciences in china..

[47] Liu M, Li D (2011). Effects of salt stress on seed germination and seedling growth of kenaf. J Henan Inst Sci Technol.

[48] Zhang J, Pan F, Liao X (2011). Response of hybrid kenaf seeding to salt stress Journal of Huazhong Agricultural University. J Huazhong Agric Univ.

[49] Qi JM, Jiang HQ, Chen MX (2012). Differential protein analysis of Kenaf leaves under drought stress. Scientia Agricultura Sinica..

[50] Li H, Li D, Chen A (2017). RNA-seq for comparative transcript profiling of kenaf under salinity stress. J Plant Res.

[51] Li H, Li D, Chen A (2017). Cloning and expression characteristics of HcWD40-1 gene under salt and drought stress in Kenaf (*Hibiscus cannabinus*). J Agric Biotechnol.

[52] Jin G, An X, Luo X (2018). The EST-SSR markers of the response gene of kenaf under drought stress. Mol Plant Breed..

[53] Luo M, Cheng K, Xu Y (2017). Plant responses to abiotic stress regulated by histone deacetylases. Front Plant Sci..

[54] Luo M, Wang YY, Liu X (2012). HD2C interacts with HDA6 and is involved in ABA and salt stress response in *Arabidopsis*. J Exp Bot.

[55] Sridha S, Wu KQ (2006). Identification of AtHD2C as a novel regulator of abscisic acid responses in *Arabidopsis*. Plant J..

[56] Han Z, Yu H, Zhao Z (2016). AtHD2D gene plays a role in plant growth, development, and response to abiotic stresses in *Arabidopsis thaliana*. Front Plant Sci..

[57] Kim JM, To TK, Matsui A (2017). Acetate-mediated novel survival strategy against drought in plants. Nat Plants..

[58] Guo JE, Hu Z, Guo X (2017). Molecular characterization of nine tissue-specific or stress-responsive genes of histone deacetylase in Tomato (*Solanum lycopersicum*). J Plant Growth Regul.

