# Supplementary material for: Molecular cloning and subcellular localization of six HDACs and their roles in response to salt and drought stress in kenaf (Hibiscus cannabinus L.)
Source: Biol Res. 2019 Apr 6;52:20. doi: 10.1186/s40659-019-0227-6 (PMC6451785; doi:10.1186/s40659-019-0227-6)
Supplement: Supplementary file 1 — Additional file 1: Table S1. Description of primers used in the study. [file 40659_2019_227_MOESM1_ESM.docx]

Table S1 Description of primers used in the study

| Primers | Base sequence 5’ to 3’ | Description |
| --- | --- | --- |
| *HcHDA2-F1* | *AGTTGCCACTCCCTTCATCG* | *PCR primer for full-length clone of DNA* |
| *HcHDA2-R1* | *CTCACTTATCGTATTTGGTTGCGT* |  |
| *HcHDA6-F1* | *TGGCTTTGACTCTCATCACTAAC* |  |
| *HcHDA6-R1* | *ACCTAAACCACCATACTTTCCTAA* |  |
| *HcHDA8-F1* | *AGTGAAGGGAGATACTGTTTGAGTTC* |  |
| *HcHDA8-R1* | *ATATGGCAAAACCCTAGATCAAACT* |  |
| *HcHDA9-F1* | *CTCTAAAATGGCGAACTCTTGGT* |  |
| *HcHDA9-R1* | *ATCAAATCCATCAGAGGTGCGAG* |  |
| *HcHDA19-F1* | *ATGGAAACCGGAGGGAATTC* |  |
| *HcHDA19-R1* | *AGCTTTCGGATCATCGATAGACAT* |  |
| *HcSRT2-F1* | *TAAAAAACACCAACCACCATTGGAG* |  |
| *HcSRT2-R1* | *CTGATTAGGCAAGGTAAAGTAGGTAAAG* |  |
| *HcGCN5-F1* | *CCCTACCCTCGCTGACAACA* |  |
| *HcGCN5-R1* | *TGTTCCCCTTGTAATGTGATGT* |  |
| *HcHAM1-F1* | *AACCGACACCATGGGTTCC* |  |
| *HcHAM1-R1* | *TAAGAGCAAACGAGTGCCATT* |  |
| *HcHDA6-R2* | *CTTGCCAGTTGCCACTCCCT* | *PCR primer for full-length clone of cDNA* |
| *HcHDA2-R2* | *CTCACTTATCGTATTTGGTTGCGT* |  |
| *HcHDA6-F2* | *ACTTCCTTTTTGTTTTCCTTAGGCG* |  |
| *HcHDA6-R2* | *AAACCACCATACTTTCCTAAGCCTAAT* |  |
| *HcHDA8-F2* | *AGTGAAGGGAGATACTGTTTGAGTTC* |  |
| *HcHDA8-R2* | *ATATGGCAAAACCCTAGATCAAACT* |  |
| *HcHDA9-F2* | *CTCTAAAATGGCGAACTCTTGGTA* |  |
| *HcHDA9-R2* | *TATGTAGTCTCTCACCTCGCACCT* |  |
| *HcHDA19-F2* | *ATGGAAACCGGAGGGAATTC* |  |
| *HcHDA19-R2* | *AGCTTTCGGATCATCGATAGACAT* |  |
| *HcSRT2-F2* | *TAAAAAACACCAACCACCATTGGAG* |  |
| *HcSRT2-R2* | *CTAATCAGCTTATCGCAGAGCAGGG* |  |
| *HcGCN5-F2* | *AATAATGGGAAGGAGGGG* |  |
| *HcGCN5-R2* | *TGTTCCCCTTGTAATGTGATGT* |  |
| *HcHAM1-F2* | *TCCATAGACACGCCGACGATTA* |  |
| *HcHAM1-R2* | *AGAGCAAACGAGTGCCATTATC* |  |
| *RT-HcHDA2-F* | *AGCATACCTGAATAGCCTGAAGAAC* | *primer for qPCR* |
| *RT-HcHDA2-R* | *AAACCCTCCTCCAACATTGATAGC* |  |
| *RT-HcHDA6-F* | *TAGAAGAGGCATTTTACACCACTGAC* |  |
| *RT-HcHDA6-R* | *ATCATCATCCATCCCATCATTCAAGG* |  |
| *RT-HcHDA8-F* | *CGAGAGATTGGACGGACAGTTC* |  |
| *RT-HcHDA8-R* | *GGGATGGAAGATTGAGCACACC* |  |
| *RT-HcHDA9-F* | *AGCGTTTACTTTGGTCCTAATCATCC* |  |
| *RT-HcHDA9-R* | *CGAACAAGTGCTGCGTATCTGG* |  |
| *RT-HcHDA19-F* | *CGGAATGTCGCTCGCTGTTG* |  |
| *RT-HcHDA19-R* | *TCGTGTTGAGGCATCTTGTCG* |  |
| *RT-HcSRT2-F* | *GAGAGTCACGAACACCTACGAAC* |  |
| *RT-HcSRT2-R* | *CTAATTCCCGCTCCAGTCAATACC* |  |
| *RT-HcGCN5-F1* | *AGGAAGAAGCAGGAAGACTCAAG* |  |
| *RT-HcGCN5-R1* | *CCATCACAAGGCGAACAATATACTC* |  |
| *RT-HcHAM1-F1* | *TGTATCCTCACCCTTCCTCCATATC* |  |
| *RT-HcHAM1-R1* | *GTGTGCCAACTTTCCCTTCTTTC* |  |
| *H3(Histone3)-F* | *GTGGAGTCAAGAAGCCTCACAG* |  |
| *H3(Histone3)-R* | *ATGGCTCTGGAAACGCAAA* |  |
| *18S(18S rRNA)-F* | *AGAAACGGCTACCACATC* |  |
| *18S(18S rRNA)-R* | *TACTCATTCCAATTACCAGACTC* |  |
| *ACT3(Actin 3)-F* | *GTGAGGATATTCAACCCCTTGTCT* |  |
| *ACT3(Actin 3)-R* | *CATCTTTCTGTCCCATACCAACC* |  |
